# Amplitude das Ondas F como Preditor de Tromboembolismo e de Sucesso da Cardioversão Elétrica em Pacientes com Fibrilação Atrial Persistente

**DOI:** 10.36660/abc.20210410

**Published:** 2022-09-15

**Authors:** Renan Teixeira Campelo, Luciana Armaganijan, Dalmo A. R. Moreira, Matheus Kiszka Scheffer, Guilherme Dagostin de Carvalho, João Italo Dias França

**Affiliations:** 1 Instituto Dante Pazzanese de Cardiologia São Paulo SP Brasil Instituto Dante Pazzanese de Cardiologia , São Paulo , SP – Brasil

**Keywords:** Fibrilação Atrial, Eletrocardiografia, Tromboembolia, Eletrofisiologia

## Abstract

**Fundamento:**

A fibrilação atrial (FA) é classificada, de acordo com a amplitude das ondas fibrilatórias (f), em ondas finas (FAf) e ondas grossas (FAg).

**Objetivos:**

Correlacionar a amplitude das ondas f com variáveis clínicas, laboratoriais, eletrocardiográficas e ecocardiográficas que indiquem alto risco de tromboembolismo e avaliar o seu impacto no sucesso da cardioversão elétrica (CVE).

**Métodos:**

Estudo retrospectivo, observacional, que incluiu 57 pacientes com FA não valvar persistente submetidos a CVE. A amplitude máxima das ondas f foi aferida na derivação V1. FAg foi definida quando f≥1,0 mm e FAf quando f<1,0mm. Os achados foram correlacionados com as variáveis indicadas. Valores de p<0,05 foram considerados estatisticamente significativos.

**Resultados:**

FAg (n=35) associou-se a maior sucesso na CVE (94,3% vs. 72,7%, p=0,036) mesmo após ajuste para variáveis como idade e IMC (p=0,026, OR=11,8). Pacientes com FAf (n=22) necessitaram mais choques e maior energia para reversão ao ritmo sinusal (p=0,019 e p=0,027, respectivamente). Não houve associação significativa entre a amplitude das ondas f e parâmetros clínicos, ecocardiográficos e laboratoriais.

**Conclusões:**

A amplitude de f não se associou a parâmetros ecocardiográficos, clínicos e laboratoriais que indicam alto risco de tromboembolismo. FAg associou-se a maior chance de sucesso na reversão ao ritmo sinusal por meio da CVE. Maior número de choques e energia foram necessários para reversão ao ritmo sinusal em pacientes com FAf.

## Introdução

A fibrilação atrial (FA) é classificada, de acordo com a amplitude das ondas fibrilatórias (f), em: FA de ondas finas (FAf) e FA de ondas grossas (FAg). Grande controvérsia existe quanto ao valor da amplitude de f como marcador para inferir riscos e contribuir no direcionamento de estratégias terapêuticas em pacientes com FA. ^1 - 4^

O objetivo deste estudo foi avaliar a relação entre a amplitude das ondas f e o risco de tromboembolismo determinado por parâmetros clínicos, laboratoriais, eletrocardiográficos e ecocardiográficos, assim como a avaliação do seu impacto no resultado da cardioversão elétrica (CVE) em pacientes com FA não valvar (FANV) persistente.

## Métodos

Trata-se de um estudo retrospectivo, observacional, com base na análise de prontuários de 57 pacientes, aprovado pelo Comitê de Ética em Pesquisa local.

Pacientes de ambos os sexos, portadores de FANV persistente (duração >7 dias, não revertidos previamente) submetidos a CVE com ou sem sucesso e que possuíam eletrocardiograma (ECG) pré e pós CVE (realizados imediatamente antes e após 1 hora da CVE, respectivamente) foram incluídos na análise.

Constituíram critérios de exclusão: flutter atrial, pacientes que apresentaram cardioversão química e prontuários não identificados ou ECGs pré/pós CVE extraviados ou com qualidade técnica comprometida.

### Análise eletrocardiográfica

**Figura 1:**
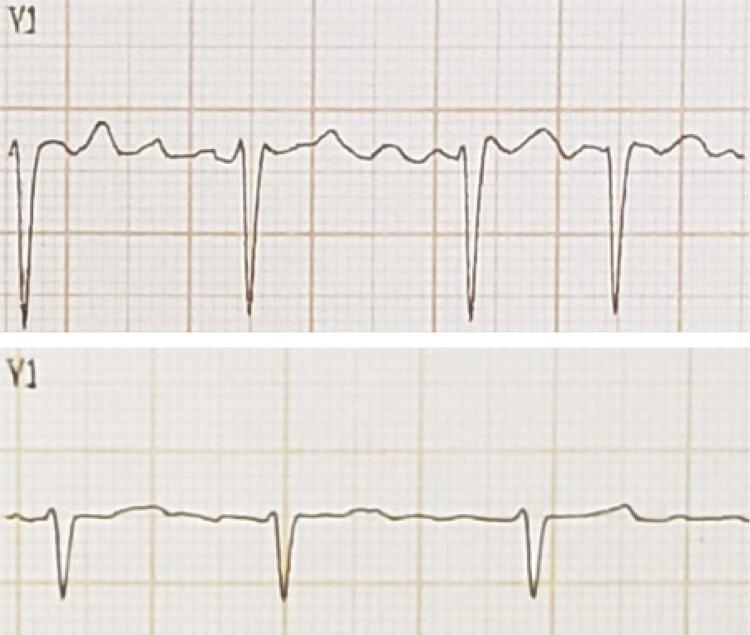
Subtipos de FA com base na amplitude das ondas f em V1. No topo, FAg. Abaixo, FAf. Fonte: arquivo pessoal.

**Figura 2:**
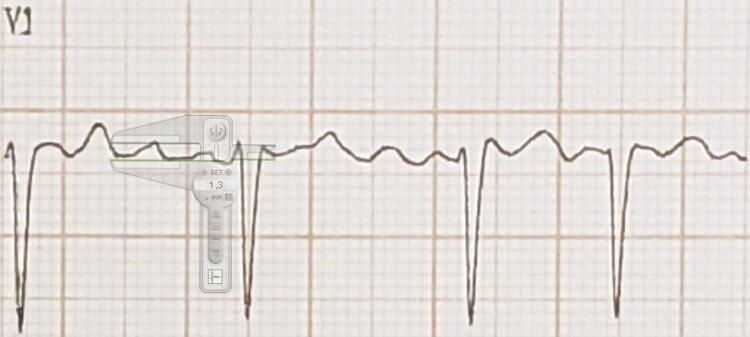
Cálculo da amplitude da onda f, partindo do pico ao vale. Fonte: arquivo pessoal.

Os ECGs pré e pós CVE, registrados com velocidade de 25mm/s, foram digitalizados. A aferição da amplitude das ondas f foi realizada com auxílio do programa *Cardio Calipers 3.3* na derivação V1. A FA foi classificada de acordo com a amplitude das ondas f em FAg, quando a amplitude máxima era ≥ 1,0mm, e FAf quando <1,0mm, medida pela deflexão máxima da onda por técnica previamente descrita ( Figura 1 ). ^5^ A amplitude máxima da onda f em V1 foi calculada com ampliação do sinal em até 10x para melhor precisão ( Figura 2 ). Foram identificadas sempre dentro do intervalo T-QRS, atentando-se para distinção correta das ondas U e ondas T. As medições foram feitas por dois examinadores independentes e cegos para os resultados do ecocardiograma transesofágico (ECOTE) e da CVE.

A duração da onda p em DII assim como a força terminal da onda p em V1 (índice de Morris) foram analisadas no ECG pós CVE, conforme técnica descrita por Peter et al. ^5^

### Realização e análise do ECOTE

O ECOTE foi realizado com ecocardiógrafo *General Eletric* com transdutor transesofágico. A aquisição das imagens seguiu a orientação da seção de ecocardiografia institucional e foi baseada nas diretrizes atuais. ^6^

Dados como tamanho e volume do átrio esquerdo (AE), fração de ejeção, presença de trombo/contraste espontâneo e velocidade de fluxo no apêndice atrial esquerdo (AAE) foram obtidos. Contraste espontâneo foi definido pela presença de “fumaça” em redemoinho na cavidade atrial, e classificado em discreto (quando vista apenas com grande ganho de sinal) e significativo (quando ocupava grande parte da cavidade atrial e visualizada mesmo com baixo ganho do sinal). Trombo atrial foi definido como massa intracavitária circunscrita, uniformemente consistente e ecorrefletiva, diferente do endocárdio atrial e da musculatura pectinada, e presente em mais de um plano de imagem.

### Realização e análise da CVE

A prescrição de fármacos antiarrítmicos por pelo menos uma semana antes da CVE foi permitida. Os pacientes também poderiam estar em uso de medicações coadjuvantes a depender das condições clínicas subjacentes e do controle da resposta ventricular.

Os choques foram realizados por médico assistente, cego para o resultado do ECOTE contanto que o paciente estivesse em uso de anticoagulante oral de ação direta (DOAC) ou antagonista de vitamina K (INR alvo entre 2-3) por pelo menos 3 semanas. Utilizou-se cardioversor de corrente contínua bifásica com pás colocadas na região anterior do tórax (segundo espaço intercostal direito) e linha hemiclavicular esquerda (sexto espaço intercostal). Os choques eram sincronizados com o pico da onda R e realizados com intensidades crescentes de energia. O protocolo era interrompido após restabelecimento do ritmo sinusal ou após se encerrarem as aplicações das cargas.

Em caso de recorrência imediata, o procedimento era repetido seguindo o mesmo protocolo. Considerado o insucesso, era realizado apenas o controle da resposta ventricular. Sucesso foi definido como a manutenção do ritmo sinusal por pelo menos 1 hora após o procedimento. A anticoagulação oral foi mantida por no mínimo 4 semanas após a CVE.

### Análise estatística

As variáveis quantitativas foram expressas pela média e desvio padrão ou mediana e intervalo interquartil (IIQ), conforme normalidade dos dados, e as variáveis categóricas pela frequência absoluta e porcentagem.

Para a análise de diferença entre os grupos, utilizou-se o teste *t* -Student para amostras independentes ou o teste não-paramétrico de Mann-Whitney para variáveis quantitativas (dependendo da suposição de normalidade dos dados testados pelo teste de Kolmogorov-Smirnov). Nas variáveis categóricas empregou-se o teste Exato de Fisher.

Para avaliar o poder discriminativo da amplitude máxima de f medida em V1 no sucesso do procedimento, ajustou-se uma curva ROC. Para a determinação do ponto de corte foi considerado o critério de Youden.

O método de Regressão Logística foi usado para ajuste univariado e multivariado da f máxima como preditora do sucesso na CVE. No modelo multivariado foram incluídas as variáveis explicativas com p valores <0,10 na análise univariada ou na comparação dos grupos FAg e FAf.

O coeficiente de correlação de concordância (CCC) e o C.b ( *correct bias* ) foram utilizados para medir a concordância intraobservador e interobservador, respectivamente.

O tamanho da amostra foi calculado com base na avaliação dos primeiros 20 pacientes incluídos no estudo. Destes, 2 apresentaram insucesso na CVE (10%) e 18 tiveram sucesso (90%), com médias de f-máx em V1 respectivamente iguais a 0,45 e 1,01 e desvio padrão comum igual a 0,37. Considerando o nível de significância de 5%, poder do teste de 90% e alocação de 9 para 1 (supondo que a cada 10 pacientes 9 têm sucesso), para detectar uma diferença de 0,56 na f-máx em V1 na comparação de casos com sucesso e insucesso, um total de 53 casos seria necessário. O cálculo foi feito com o programa Stata/SE v.14.1. StataCorpLP, USA.

Os dados foram analisados no programa SPSS versão 19.0. O nível de significância adotado foi de 5%.

## Resultados

**Figura 3:**
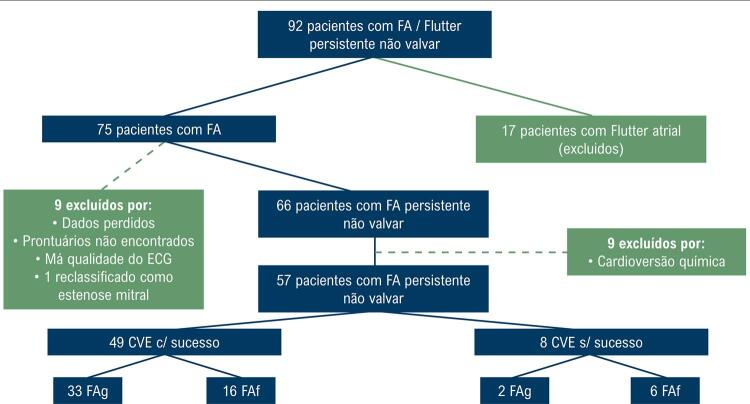
Fluxograma dos pacientes incluídos e excluídos do estudo. FA: fibrilação atrial; ECG: eletrocardiograma; CVE: cardioversão elétrica; FAg: fibrilação atrial de ondas finas; FAf: fibrilação atrial de ondas grossas.

Dos 92 pacientes selecionados, apenas 57 atingiram os critérios de elegibilidade. Em 8 (14%; IC95%: 5,0%-23,1%) deles não se obteve sucesso na CVE ( Figura 3 ).

### Características clínicas

A maioria dos pacientes era idosos do sexo masculino. A comorbidade mais frequente foi hipertensão e mais da metade apresentava CHA _2_ DS _2_ VASc≥2. O anticoagulante mais utilizado foi a varfarina e cinco pacientes estavam sob uso de DOAC. A maioria foi pré-tratada com amiodarona e grande parte vinha em uso de betabloqueador ( Tabela 1 ).

**Tabela 1:** Características gerais e com base na amplitude de f máxima em V1

Variável	População geral N=57	FAg (f-máx V1 *≥* 1mm) N=35 (61,4%)	FAf (f-máx V1<1mm) N=22 (38,6%)	p*
Idade (anos)	61,53±10,86	61,43±12,41	61,68±8,07	0,933
Sexo masculino	40 (70,2%)	23 (65,7%)	17 (77,3%)	0,391
Peso (Kg)	86,1±22,8	81,0±18,13	94,23±27,31	0,032
Superfície corpórea (m ^2^ )	2,79±0,28	1,90±0,25	2,05±0,30	0,054
IMC (Kg/m ^2^ )	29,77±6,04	28,44±4,62	31,88±7,42	0,061
HAS	47 (82,5%)	27 (77,1%)	20 (90,9%)	0,287
DM	14 (24,6%)	8 (22,9%)	6 (27,3%)	0,758
DAC	9 (15,8%)	4 (11,4%)	5 (22,7%)	0,286
ICC	4 (7,0%)	4 (11,4%)	0 (0%)	0,151
AVC	7 (12,3%)	4 (11,4%)	3 (13,6%)	1
IVP	4 (7,0%)	3 (8,6%)	1 (4,5%)	1
CHA _2_ DS _2_ VASc	2 (1–3)	2 (1–3)	2 (1–3)	0,880
0	5 (8,8%)	3 (8,6%)	2 (9,1%)	
1	17 (29,8%)	11 (31,4%)	6 (27,3%)	
≥2	35 (61,4%)	21 (60%)	14 (63,6%)	
Duração da FA (dias)	210 (90-365)	210 (90–365)	225 (60–365)	0,938
Varfarina	52 (91,2%)	31 (88,6%)	21 (95,5%)	0,639
DOAC	5 (8,8%)	4 (11,4%)	1 (4,5%)	
Amiodarona	54 (94,7%)	34 (97,1%)	20 (90,9%)	0,553
Propafenona	3 (5,3%)	1 (2,9%)	2 (9,1%)	0,553
Betabloqueador	42 (73,7%)	26 (74,3%)	16 (72,7%)	1
FEVE (%)	55,44±11,55	54,09±13,64	57,59±6,85	0,948
Diâmetro AE (mm)	46,91±5,14	47,20±5,31	46,45±4,94	0,599
Volume indexado AE (ml/m ^2^ )	52,38±13,73	53,57±14,57	50,38±12,28	0,405
Velocidade fluxo AAE (cm/s)	30,26±9,69	28,71±8,99	32,83±10,49	0,125
Contraste espontâneo	32 (56,1%)	20 (57,1%)	12 (54,5%)	1
Trombo AE	5 (8,8%)	4 (11,4%)	1 (4,5%)	0,639
PRO-BNP pré-CVE	1090 (595-1960)	1280 (565–2450)	870 (626-1344)	0,254
PCR pré-CVE	0,61 (0,30–1,10)	0,50 (0,25–1,00)	1,10 (0,50–1,50)	0,070
INR pré-CVE	2,73 (2,47–3,28)	2,73 (2,50–3,29)	2,63 (2,43–3,21)	0,948
F-máxima V1 (mm)	1,11±0,51	1,41±0,41	0,64±0,16	<0,001
Índice Morris pós-CVE	29 (58%)	21 (63,6%)	8 (47,1%)	0,366
Duração p-DII pós CVE (ms)	128,41±26,42	130,38±20,54	124,35±36,16	0,542

Variáveis quantitativas descritas por média ± desvio padrão ou mediana (intervalo interquartil); variáveis categóricas descritas por frequência (percentual); *Associação entre FAg e FAf: teste t de Student para amostras independentes ou teste não-paramétrico de Mann-Whitney (variáveis quantitativas); teste exato de Fisher (variáveis categóricas); p<0,05. FAg: fibrilação atrial de ondas grossas; FAf: fibrilação atrial de ondas finas; IMC: índice de massa corpórea; HAS: hipertensão: DM: diabetes mellitus; DAC: doença arterial coronariana; ICC: insuficiência cardíaca congestiva; AVC: acidente vascular cerebral; IVP: insuficiência vascular periférica; FA: fibrilação atrial; DOAC: anticoagulante oral de ação direta; FEVE: fração de ejeção do ventrículo esquerdo; AE: átrio esquerdo; AAE: apêndice atrial esquerdo; CVE: cardioversão elétrica; PCR: Proteína C reativa; INR: relação normalizada internacional.

### Características laboratoriais e ecocardiográficas

A FEVE média da maioria dos pacientes era preservada. Apenas 7 (12,3%) apresentavam valores < 40%. A despeito da anticoagulação, trombo e/ou contraste espontâneo significativo no AE foram observados em 35 pacientes. Os valores médios de PRO-BNP e PCR (Proteína C reativa) pré-CVE foram elevados. ( Tabela 1 ).

### Características eletrocardiográficas

A amplitude das ondas f-máxima medidas em V1 variou de 0,3 a 2,9mm. O índice de Morris esteve alterado na maioria dos pacientes que restauraram o ritmo sinusal e a duração média da onda P em DII nesses pacientes era elevada ( Tabela 1 ).

### Características com base na amplitude de f

Não se observaram diferenças entre os grupos quanto às características clínicas e ecocardiográficas, exceto o peso. O grupo FAf era composto por pacientes com valores de peso mais elevados em comparação ao grupo FAg ( Tabela 1 ).

### Sucesso na CVE

**Figura 4:**
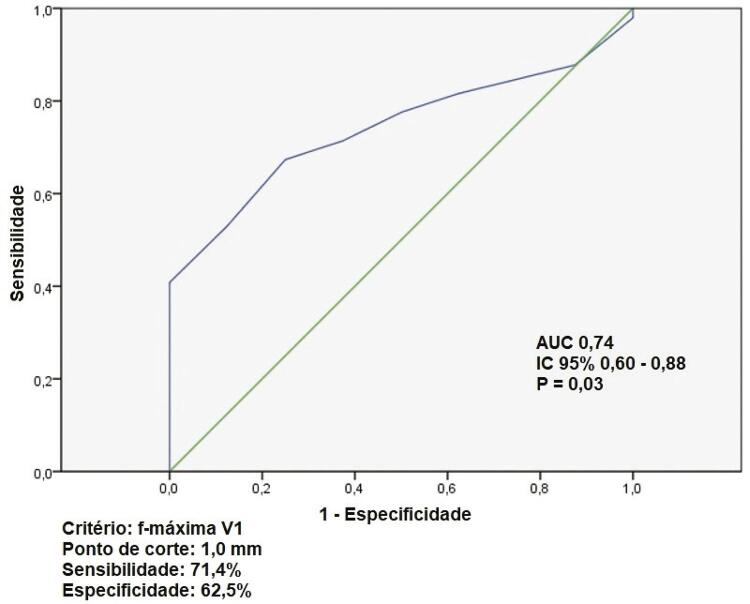
Forest plot com OR e IC 95% dos parâmetros clínicos, ecocardiográficos e eletrocardiográficos relacionados ao sucesso na cardioversão elétrica (análise univariada). FAg: fibrilação atrial de ondas grossas; IMC: índice de massa corpórea; HAS: hipertensão: DM: diabetes mellitus; DAC: doença arterial coronariana; ICC: insuficiência cardíaca congestiva; AVC: acidente vascular cerebral; IVP: insuficiência vascular periférica; FA: fibrilação atrial; FEVE: fração de ejeção do ventrículo esquerdo; AE: átrio esquerdo; AAE: apêndice atrial esquerdo; PCR: Proteína C reativa;

Nenhum parâmetro interferiu no sucesso da CVE. Apenas a presença de FAg favoreceu esse desfecho (94,3% vs 72,7%, p=0,036; OR 6,17; IC 95% 1,21-34,5) ( Tabela 2 e Figura 4 ).

**Tabela 2:** Associações entre parâmetros ecocardiográficos, laboratoriais e eletrocardiográficos com o sucesso na cardioversão elétrica

Variável	N (57)	Sucesso N=49 (86%)	Insucesso N=8 (14%)	p*
Idade (anos)		62,55±10,40	55,25±12,22	0,088
Sexo Feminino	17	15 (88,2%)	2 (11,8%)	
Masculino	40	34 (85%)	6 (15%)	0,748
Peso (Kg)		84,82±23,39	94,00±18,39	0,296
Superfície corpórea (m ^2^ )		1,94±0,29	2,07±0,23	0,244
IMC (Kg/m ^2^ )		29,46±6,03	31,65±6,17	0,344
HAS	47	42 (89,4%)	5 (10,6%)	0,126
DM	14	12 (85,7%)	2 (14,3%)	0,975
DAC	9	9 (100%)	0 (0%)	0,332**
ICC	4	4 (100%)	0 (0%)	1**
AVC	7	6 (85,7%)	1 (14,3%)	0,977
IVP	4	4 (100%)	0 (0%)	1**
CHA _2_ DS _2_ VASc		2 (1–3)	1 (0,5–3)	0,200
0	5	3 (60%)	2 (40%)	
1	17	14 (82,3%)	3 (17,6%)	
≥2	35	32 (91,4%)	3 (8,6%)	
Duração da FA (dias)		210 (100–370)	135 (75–270)	0,190
Amiodarona	54	48 (88,9%)	6 (11,1%)	-
Propafenona	3	1 (33,3%)	2 (66,7%)	-
Betabloqueador	42	37 (88,1%)	5 (11,9%)	0,443
FEVE (%)		55,67±11,68	54±11,39	0,702
Diâmetro AE (mm)		47,24±5,11	44,88±5,19	0,229
Volume indexado AE (ml/m ^2^ )		52,67±14,12	50,29±11,32	0,665
Velocidade fluxo AAE (cm/s)		29,43±8,81	35,25±13,54	0,125
Contraste espontâneo	32	29 (90,6%)	3 (9,4%)	0,262
Trombo AE	5	4 (80%)	1 (20%)	0,690
PRO-BNP pré-CVE		1195 (564–2005)	776 (649–1272)	0,607
PCR pré-CVE		0,60 (0,25–1,10)	0,71 (0,55–1,50)	0,142
INR pré-CVE		2,72 (2,50–3,29)	2,72 (2,24–2,95)	0,836
F-máxima V1 (mm)		1,15±0,50	0,85±0,47	0,118
≥1 (FAg)	35	33 (94,3%)	2 (5,7%)	
<1 (FAf)	22	16 (72,7%)	6 (27,3%)	0,036

Variáveis quantitativas descritas por média ± desvio padrão ou mediana (intervalo interquartil); variáveis categóricas descritas por frequência (percentual); *Modelos de Regressão Logística univariados e teste de Wald, p<0,05. ** Teste exato de Fisher, p<0,05. FAg: fibrilação atrial de ondas grossas; FAf: fibrilação atrial de ondas finas; IMC: índice de massa corpórea; HAS: hipertensão: DM: diabetes mellitus; DAC: doença arterial coronariana; ICC: insuficiência cardíaca congestiva; AVC: acidente vascular cerebral; IVP: insuficiência vascular periférica; FA: fibrilação atrial; FEVE: fração de ejeção do ventrículo esquerdo; AE: átrio esquerdo; AAE: apêndice atrial esquerdo; CVE: cardioversão elétrica; PCR: Proteína C reativa; INR: relação normalizada internacional.

**Figura 5:**
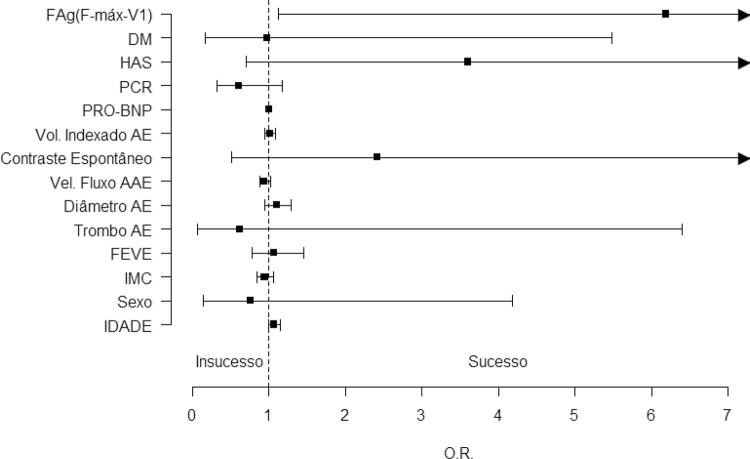
Curva ROC da máxima amplitude de f em V1 como preditor de sucesso da cardioversão elétrica.

Ajustou-se uma curva operacional com vistas a determinar o melhor ponto de corte da f máxima em V1 associada ao sucesso da CVE. O valor de 1,0mm foi o que apresentou melhor acurácia ( Figura 5 ).

**Figura 6:**
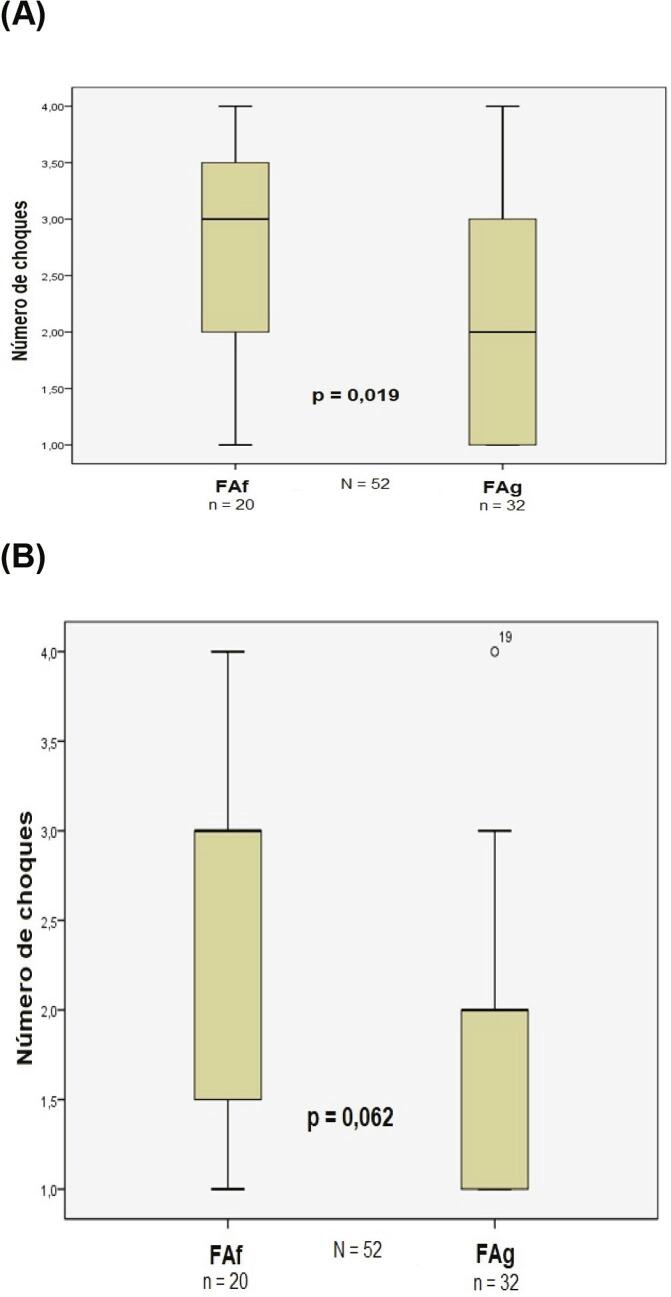
Número de choques aplicados em cada grupo (A) e número de choques necessários (B) para reversão ao ritmo sinusal em ambos os grupos. FAg: fibrilação atrial de ondas grossas; FAf: fibrilação atrial de ondas finas.

**Figura 7:**
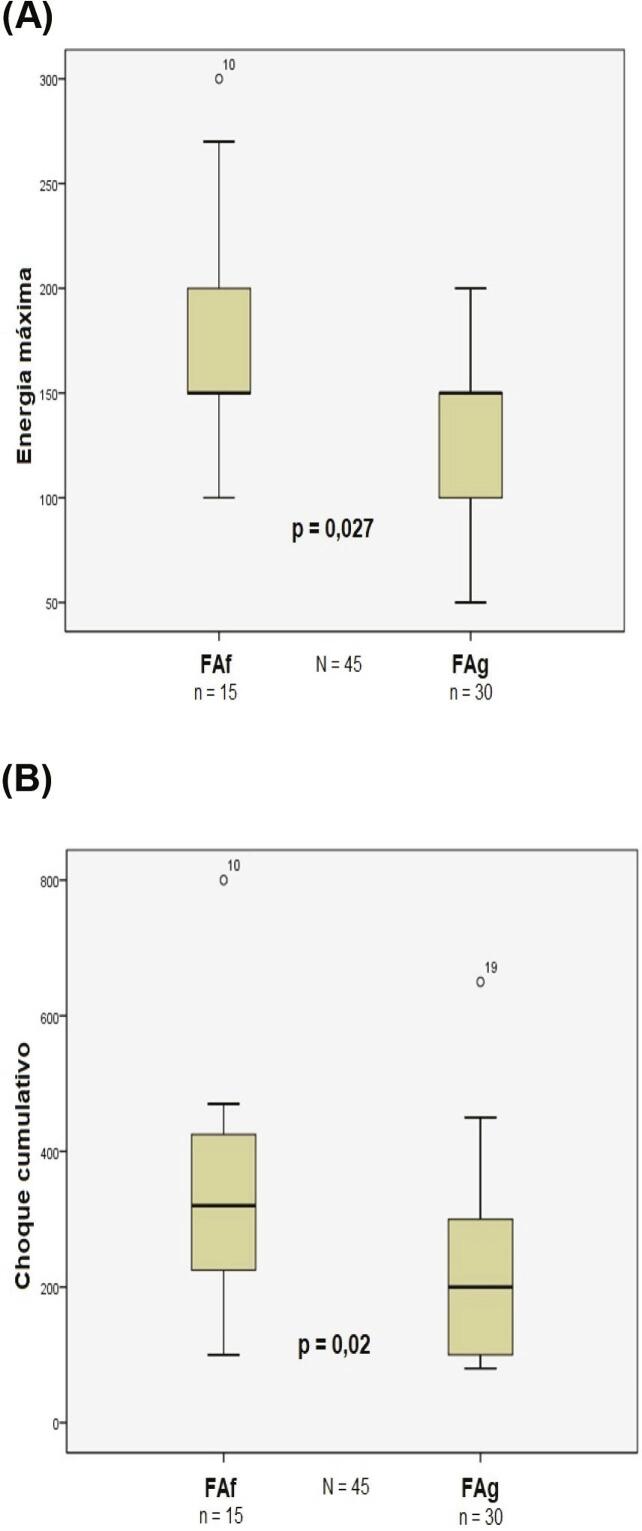
Energias máxima (A) e cumulativa (B) necessárias para reversão ao ritmo sinusal em ambos os grupos. FAg: fibrilação atrial de ondas grossas; FAf: fibrilação atrial de ondas finas.

Pacientes com FAf receberam, em mediana, 3 (2–3,5) choques comparados com 2 (1–3) no grupo com FAg (p=0,019). Quando analisados apenas os que obtiveram sucesso na CVE, o grupo FAf necessitou maior número de choques para reversão ao ritmo sinusal [3 (1–3) vs 2 (1–2), p=0,064] ( Figura 6 ). Da mesma forma, as energias máxima e cumulativa (soma das cargas) utilizadas para reversão ao ritmo sinusal foram maiores no grupo FAf [150J (150–200J) vs 150J (100–150J), p=0,027 e 320J (200–450J) vs 200J (100–300J), p=0,020; respectivamente] ( Figura 7 ).

Na análise multivariada, a presença de FAg associou-se ao sucesso da CVE (B=2,470, p=0,026), independentemente de idade e IMC, favorecendo em 11,8 vezes o evento reversão para o ritmo sinusal.

### Variabilidades intra e interobservador

O cálculo da variabilidade intraobservador mostrou CCC e Cb de 0,90 e 0,98 para f máxima em V1, respectivamente. Da mesma forma, os valores de CCC e Cb para variabilidade interobservador foram de 0,90 e 0,98, respectivamente.

## Discussão

No presente estudo, a amplitude das ondas f não se associou com parâmetros clínicos, laboratoriais e ecocardiográficos sugestivos de maior risco de tromboembolismo. No entanto, contribuiu na predição da reversão ao ritmo sinusal por meio da CVE.

Diversos fatores que proporcionam o aumento do risco de tromboembolismo em pacientes com FA estão relacionados entre si dificultando a análise individual como fatores independentes. Na amostra estudada, todos os pacientes estavam sob anticoagulação plena (maioria sob uso de varfarina e com controle rigoroso do INR pré-CVE). Nas publicações que propuseram avaliar a correlação entre a amplitude de f e tromboembolismo, nenhuma amostra era composta por 100% dos pacientes anticoagulados adequadamente. No estudo de Icen et al., ^7^ por exemplo, 89% dos pacientes estavam em uso de anticoagulantes e relatos de eventos tromboembólicos foram descritos em pacientes fora da faixa de anticoagulação. No estudo por Nakagawa et al. ^8^ apenas 54% dos pacientes estavam sob anticoagulação plena. Já na pesquisa realizada por Yamamoto et al., ^9^ apenas aqueles com contraste espontâneo ou trombo tinham indicação de anticoagulação (75%). Todos esses estudos apresentavam diferenças percentuais quanto à terapêutica anticoagulante entre os grupos definidos com base na amplitude de f.

Apesar da anticoagulação adequada em todos os pacientes da amostra, 56,1% exibiam contraste espontâneo significativo e 8,8% possuíam trombo no AE, evidenciando que outros mecanismos, não tratados pela anticoagulação, ainda estariam presentes aumentando o risco de tromboembolismo. Mesmo assim, não houve correlação significativa desses achados com a amplitude das ondas f, fato este também encontrado por Nakagawa et al. ^8^ A presença de contraste espontâneo também não se associou à amplitude de f na análise de Yamamoto et al., ^9^ entretanto, os autores reportaram maior percentual de pacientes com trombo no AE e eventos tromboembólicos no grupo FAf, o que pode ser explicado pela menor porcentagem de pacientes sob anticoagulação nesse grupo durante o seguimento.

Contrariando esses achados, Li et al. ^10^ encontraram relação entre FAg e a presença de contraste espontâneo, trombo no AE e disfunção no AAE. Apesar de ambos os grupos serem mais uniformes em relação à terapia anticoagulante, os autores não reportaram sobre as diferenças no escore CHA _2_ DS _2_ VASc entre os grupos, o que poderia estar influenciando na variação de trombogênese entre eles. Além disso, havia uma diferença de um mês entre a realização do ECOTE e do ECG, o que pode ter contribuído com os achados. No presente estudo, pacientes com FAg e FAf apresentavam valores semelhantes para CHA _2_ DS _2_ VASc, idade, IMC e outros parâmetros clínicos. Todos estavam sob anticoagulação e o ECOTE e ECG foram realizados no mesmo momento.

Na presente amostra, não foram incluídos pacientes com estenose mitral. O motivo foi que a estase sanguínea causada pela obstrução do fluxo na valva mitral predispõe a alterações ecocardiográficas, que no caso estariam mais relacionados ao próprio fator obstrutivo do que com a amplitude das ondas f. Particularmente, portadores de estenose apresentam AE dilatados e hipertróficos, com aumento da pressão intracavitária atrial. Uma vez que pacientes com estenose mitral são, na sua maioria, de etiologia reumática, apresentam idade mais jovem e menos comorbidades, apesar de maiores, os átrios são menos remodelados eletricamente gerando circuitos reentrantes maiores, que se expressam por um vetor resultante mais proeminente no ECG (FAg). ^11 - 13^

Quanto ao tamanho do AE, não foram observadas diferenças significativas entre os grupos e esses achados estão de acordo com diversas outras publicações. ^8 , 10 , 14 , 15^ Isso se dá pelo fato de que a dilatação atrial não traduz de maneira fidedigna o grau de remodelamento elétrico, estrutural e histológico sofrido pelo átrio. Em ambos os grupos, os valores encontrados para diâmetro e volume atriais eram elevados o que diminuiu a influência dessa variável sobre as ondas f.

Já quanto a avaliação do AAE, observamos redução da velocidade de fluxo do AAE em ambos os grupos, porém, sem diferenças entre eles. Apesar de ser uma estrutura anexa ao AE, o AAE contribui para a atividade elétrica e mecânica atrial. Correlacionar suas alterações com a amplitude de f é um desafio uma vez que muitos fatores podem influenciar na sua performance, tais como: a morfologia, função (medida pela velocidade de fluxo ou fração de ejeção), grau de fibrose e a área do orifício de entrada. ^16^

Li et al. ^10^ mostraram correlação entre FAg, em pacientes com FANV, e baixa velocidade de fluxo no AAE, resultados contraditórios aos de Yamamoto et al. ^9^ e Nakagawa et al. ^8^ que mostraram associação com o grupo FAf. Por outro lado, Blackshear et al., ^14^ ao avaliarem 53 pacientes envolvidos no *SPAF III* , não encontraram associação entre a amplitude de f e a velocidade de fluxo no AAE, justificando o achado por discordância temporal entre o ECG e o ECOTE. No presente estudo, houve correlação temporal satisfatória entre o ECG e o ECOTE e, mesmo assim, não se demonstrou associação significativa com a amplitude de f.

Da mesma forma, as variáveis clínicas não mostraram associação com a amplitude da onda f. Os grupos continham pacientes com idades, sexo e escore CHA _2_ DS _2_ VASc semelhantes, conferindo maior homogeneidade e diminuindo a interferência sobre outras variáveis. Dado que a amplitude de f traduz informações sobre o remodelamento atrial, esperaríamos que pacientes com FAf apresentassem maiores escores CHA _2_ DS _2_ VASc, duração de FA e idades mais elevadas. Nesse contexto, o tamanho da amostra pode ter sido um fator limitante.

Dentre as comorbidades apresentadas, a hipertensão foi mais prevalente no grupo FAf (90,9% vs. 77,1%) concordando com achados de Yilmaz et al. ^17^ e Icen et al. ^7^ em pacientes com FANV. O IMC também tendeu a ser maior no grupo FAf o que pode ter sido um fator confundidor, uma vez que esta relação não foi descrita na literatura.

Em relação aos dados laboratoriais, no grupo FAf, os valores de PCR foram mais elevados, apesar de não ser estatisticamente significativos. Dado que este representa a presença de processo inflamatório e está relacionado com o risco de AVC e prognóstico em pacientes com FA, é plausível esperar-se valores mais elevados em pacientes com FAf, já que estes apresentam átrios remodelados com mais frequência em decorrência de múltiplos fatores, inclusive aqueles que geram inflamação. ^18^ Já os níveis de PRO-BNP encontraram-se elevados em ambos os grupos. Esse achado é frequente em portadores de FA e atua como marcador de cardiopatia atrial, além de ser indicativo de maior risco de AVC e morte nessa população. ^19^

No tocante a taxa de sucesso da CVE, esta foi de 86%, semelhante a estudos prévios. ^20 - 22^ No estudo de Zhao et al., ^20^ apesar da presença de FAg associar-se a maiores taxas de manutenção do ritmo sinusal após 6 semanas da CVE (72% vs. 42%), não houve diferença no sucesso imediato do procedimento entre os grupos (100% FAg vs. 94% FAf). Dados sobre doença valvar mitral, entretanto, não foram mencionados e isso justificaria a recorrência precoce da FA após CVE. ^23 , 24^ Já no presente estudo, a presença de FAg foi fator preditor independente para reversão imediata ao ritmo sinusal. Além de maiores taxas de sucesso da CVE, a presença de FAg resultou na necessidade de menor número de choques, assim como menor energia máxima e cumulativa comparada com a FAf. Isso é relevante na prática clínica pois contribui como mais um fator para a decisão de indicação ou não da CVE em pacientes com FA persistente.

É possível que a FAg esteja relacionada a presença de mais músculo viável nos átrios que acomodam circuitos de reentrada mais organizados, facilitando a anulação das frentes de onda por meio da cardioversão. A idade, o tipo de arritmia e duração da FA, fatores que influenciam nas taxas de sucesso da CVE, ^25^ não influenciaram no poder discriminatório da amplitude das ondas f por não diferirem entre os grupos formados.

Quanto à derivação analisada, escolhemos V1 por ser a que mais expressa alterações nos átrios devido à proximidade, por apresentar valores mais elevados da amplitude de f facilitando a medição, e por ter sido derivação aplicada pela maioria dos estudos publicados no tópico desde 1966.

Quanto ao ponto de corte utilizado para classificar a FA, optamos pelo valor de 1,0 mm baseado no fato de que não há diferença significativa entre os achados quando se utiliza o valor de 0,5mm e 1mm, conforme demonstrado por Peter et al., e o valor mais alto facilita sua mensuração. ^5^ A utilização de pontos de corte menores implica na utilização de técnicas mais acuradas de medição e mais erros de aferição, sendo poucos os ganhos em sensibilidade e especificidade.

A utilização de fármacos antiarrítmicos como pré-tratamento antes da CVE foi permitida para melhor estabilização da atividade elétrica atrial e como prevenção de recorrência precoce da arritmia. ^24^ O fato de a quase totalidade ter usado amiodarona diminui as interferências entre os grupos no resultado da CVE. Além disso, Nault et al. ^26^ demonstraram não haver influência de antiarrítmicos como amiodarona na amplitude de f.

É possível que o tamanho pequeno da amostra possa ter influenciado nos resultados, particularmente quanto à associação entre parâmetros ecocardiográficos e a amplitude de f. Estudos com maior número de pacientes são necessários para firmar essas relações.

## Conclusões

A amplitude de f não se associou a alterações clínicas e ecocardiográficas que sinalizam maior risco de tromboembolismo. Onda f máxima ≥1,0mm medida na derivação V1 associou-se a maior chance de sucesso na reversão ao ritmo sinusal por meio da CVE em pacientes com FANV persistente. Maior número de choques e energia foram necessários para reversão ao ritmo sinusal em pacientes com FAf comparados com FAg.
