# Supplementary material for: Amplitude das Ondas F como Preditor de Tromboembolismo e de Sucesso da Cardioversão Elétrica em Pacientes com Fibrilação Atrial Persistente
Source: Arq Bras Cardiol. 2022 Sep 15;119(5):778–88. [Article in Portuguese] doi: 10.36660/abc.20210410 (PMC9750208; doi:10.36660/abc.20210410)
Supplement: Translation

# Translation: F Wave Amplitude as a Predictor of Thromboembolism and Success of Electrical Cardioversion in Patients with Persistent Atrial Fibrillation

**Authors:** Renan Teixeira Campelo, Luciana Armaganijan, Dalmo A. R. Moreira, Matheus Kiszka Scheffer, Guilherme Dagostin de Carvalho, João Italo Dias França

**Affiliations:** 1 Brazil Instituto Dante Pazzanese de Cardiologia, São Paulo, SP – Brazil

**Keywords:** Atrial Fibrillation, Electrocardiography, Thromboembolism, Electrophysiology

## Abstract

**Background:**

Atrial fibrillation (AF) is classified according to the amplitude of fibrillatory waves (f) into fine waves (fAF) and coarse waves (cAF).

**Objectives:**

To correlate the amplitude of f waves with clinical, laboratory, electrocardiographic, and echocardiographic variables that indicate a high risk of thromboembolism and to assess their impact on the success of electrical cardioversion (ECV).

**Methods:**

Retrospective, observational study that included 57 patients with persistent non-valvular AF who underwent ECV. The maximum amplitude of f waves was measured in lead V1. cAF was defined when f ≥ 1.0mm and fAF when f < 1.0mm. The findings were correlated with the indicated variables. Values of p < 0.05 were considered statistically significant.

**Results:**

cAF (n = 35) was associated with greater success in ECV (94.3% vs. 72.7%, p = 0.036) even after adjusting for variables such as age and BMI (p = 0.026, OR = 11.8). Patients with fAF (n = 22) required more shocks and more energy to revert to sinus rhythm (p = 0.019 and p = 0.027, respectively). There was no significant association between f-wave amplitude and clinical, echocardiographic, and laboratory parameters.

**Conclusions:**

The amplitude of f wave was not associated with echocardiographic, clinical and laboratory parameters that indicate a high risk of thromboembolism. cAF was associated with a higher chance of success reverting to sinus rhythm employing ECV. A greater number of shocks and energy were required for reversion to sinus rhythm in patients with fAF.

## Introduction

Atrial fibrillation (AF) is classified, according to the amplitude of the fibrillatory waves (f), into fine wave AF (fAF) and coarse wave AF (cAF). There is great controversy regarding the value of f-amplitude as a marker for inferring risks and contributing to the direction of therapeutic strategies in patients with AF.^1 - 4^

The objective of this study was to evaluate the relationship between the amplitude of f waves and the risk of thromboembolism determined by the clinical, laboratory, electrocardiographic, and echocardiographic parameters, as well as to assess their impact on the outcome of electrical cardioversion (ECV) in patients with persistent non-valvular AF (NVAF).

## Methods

This was a retrospective, observational study based on the analysis of medical records of 57 patients approved by the local research ethics committee.

Patients of both genders with persistent NVAF (duration > 7 days, not previously reversed) undergoing ECV with or without success and who had pre and post-ECV electrocardiogram (ECG) (performed immediately before and 1 hour after ECV, respectively) were included in the analysis.

Exclusion criteria were: atrial flutter, patients who had pharmacologic cardioversion, and non-interpretable ECG.

### Electrocardiographic analysis

Pre and post ECV ECG, recorded at a speed of 25 mm/s, were digitized. F wave amplitude was measured in lead V1 using the CardioCalipers® 3.3 program. AF was classified according to the amplitude of f waves in cAF, when the maximum amplitude was ≥ 1.0mm, and fAF when < 1.0mm, measured by the maximum wave deflection by the previously described technique ( Figure 1 ).^5^ The maximum amplitude of the f (f-max) wave in V1 was calculated with signal magnification up to 10x for better accuracy ( Figure 2 ). They were always identified within the T-QRS interval, paying attention to the correct distinction between U and T waves. Measurements were performed by two independent examiners who were blinded to the results of the transesophageal echocardiogram (TEE) and ECV.

**Figure 1:** AF subtypes based on the amplitude of f waves in V1. At the top, cAF. On the bottom, fAF. Source: personal archive.

**Figure 2:** Calculation of wave f amplitude from peak to valley. Source: personal archive.

The duration of the p wave in lead II and the terminal deflection of the p wave in V1 (Morris index) were analyzed on the ECG after ECV, according to the technique described by Peter et al.^5^

### Conducting and analyzing the TEE

The TEE was performed with a *General Electric* echo with a transesophageal transducer. The acquisition of images followed the guidance of the institutional echocardiography section and was based on current guidelines.^6^

Data such as left atrium (LA) size and volume, ejection fraction (LVEF), presence of thrombus/spontaneous contrast, and left atrial appendage (LAA) flow velocity were obtained. Spontaneous contrast was defined by the presence of swirling “smoke” in the atrial cavity and classified as discrete (when seen only with high signal gain) and significant (when it occupied a large part of the atrial cavity and visualized even with low signal gain). Atrial thrombus was defined as a circumscribed, uniformly consistent and echo reflective intracavitary mass, different from the atrial endocardium and pectineal musculature and present in more than one imaging plane.

### Conducting and analyzing the ECV

Prescription of antiarrhythmic drugs for at least one week before ECV was allowed. Patients could also be on adjunctive medications depending on underlying clinical conditions and control of the ventricular response.

Shocks were performed by an attending physician, blinded to the TEE result, as long as the patient had been on a direct-acting oral anticoagulant (DOAC) or vitamin K antagonist (target INR between 2-3) for at least 3 consecutive weeks. A biphasic direct current cardioverter was used with paddles placed in the anterior chest region (second right intercostal space) and left midclavicular line (sixth intercostal space). The shocks were synchronized with the peak of the R wave and performed with increasing intensities of energy. The protocol was interrupted after sinus rhythm was reestablished or after the loading applications had ended.

In case of immediate recurrence, the procedure was repeated following the same protocol. When considering failure, the control of the ventricular response was performed. Success was defined as the maintenance of sinus rhythm for at least 1 hour after the procedure. Oral anticoagulation was continued for at least 4 weeks after ECV.

### Statistical analysis

Quantitative variables were expressed as mean and standard deviation or median and interquartile range (IQR), according to data normality, and categorical variables as absolute frequency and percentage.

To analyze the difference between groups, the *t* -Student test was used for independent samples or the non-parametric Mann-Whitney test for quantitative variables (depending on the assumption of normality of the data assessed by the Kolmogorov-Smirnov test). For categorical variables, Fisher’s exact test was used.

A ROC curve was fitted to assess the discriminative power of the maximum amplitude of f waves measured in V1 in the success of the procedure. To determine the cutoff point, the Youden criteria were considered.

A logistic regression model was used for uni and multivariable adjustment of maximum f as a predictor of success in ECV. In the multivariable model were included explanatory variables with p values < 0.10 in the univariable analysis or the comparison of the cAF and fAF groups.

The correlation coefficient of agreement (CCC) and C.b ( *correct bias* ) were used to measure intraobserver and interobserver agreement, respectively.

The sample size was calculated based on evaluating the first 20 patients included in the study. Of these, 2 were unsuccessful in ECV (10%), and 18 were successful (90%), with average f-max in V1 respectively equal to 0.45 mm and 1.01 mm (SD ± 0.37 mm). Considering the significance level of 5%, test power of 90% and allocation of 9 to 1 (assuming that in every 10 patients, 9 are successful), to detect a difference of 0.56 mm in f-max in V1 in comparison to successful and unsuccessful cases, a total of 53 cases would be required. The calculation was performed using the Stata/SE v.14.1 program, StataCorpLP, USA.

Data were analyzed using SPSS version 19.0. The significance level adopted was 5%.

## Results

Of the 92 selected patients, only 57 met the eligibility criteria. In 8 (14%; 95% CI: 5.0%-23.1%) of them, ECV was not successful ( Figure 3 ).

**Figure 3:** Study flowchart. AF: atrial fibrillation; ECG: electrocardiogram; NVAF: non-valvular AF; cAF: coarse f-waves atrial fibrillation; fAF: fine f-waves atrial fibrillation; ECV: electrical cardioversion.

### Clinical features

Patients were predominantly elderly males. The most frequent comorbidity was hypertension, and more than half had CHA_2_DS_2_VASc ≥ 2. The most used anticoagulant was warfarin, and five patients were on DOACs. Most were pre-treated with amiodarone and were using β-blockers ( Table 1 ).

**Table 1:** General characteristics and based on the maximum f amplitude in V1

Variable	General population N=57	cAF (f-max V1 ≥ 1 mm) N=35 (61.4%)	fAF (f-max V1 < 1 mm) N=22 (38.6%)	p*
Age (years)	61.53±10.86	61.43±12.41	61.68±8.07	0.933
Male gender	40 (70.2%)	23 (65.7%)	17 (77.3%)	0.391
Weight (Kg)	86.1±22.8	81.0±18.13	94.23±27.31	0.032
Body surface (m^2^)	2.79±0.28	1.90±0.25	2.05±0.30	0.054
BMI (Kg/m^2^)	29.77±6.04	28.44±4.62	31.88±7.42	0.061
Hypertension	47 (82.5%)	27 (77.1%)	20 (90.9%)	0.287
Diabetes	14 (24.6%)	8 (22.9%)	6 (27.3%)	0.758
CAD	9 (15.8%)	4 (11.4%)	5 (22.7%)	0.286
HF	4 (7.0%)	4 (11.4%)	0 (0%)	0.151
Stroke	7 (12.3%)	4 (11.4%)	3 (13.6%)	1
PAD	4 (7.0%)	3 (8.6%)	1 (4.5%)	1
CHA_2_DS_2_VASc	2 (1–3)	2 (1–3)	2 (1–3)	0.880
0	5 (8.8%)	3 (8.6%)	2 (9.1%)	
1	17 (29.8%)	11 (31.4%)	6 (27.3%)	
≥ 2	35 (61.4%)	21 (60%)	14 (63.6%)	
AF duration (days)	210 (90-365)	210 (90–365)	225 (60–365)	0.938
Warfarin	52 (91.2%)	31 (88.6%)	21 (95.5%)	0.639
DOAC	5 (8.8%)	4 (11.4%)	1 (4.5%)	
Amiodarone	54 (94.7%)	34 (97.1%)	20 (90.9%)	0.553
Propafenone	3 (5.3%)	1 (2.9%)	2 (9.1%)	0.553
β-blocker	42 (73.7%)	26 (74.3%)	16 (72.7%)	1
LVEF (%)	55.44±11.55	54.09±13.64	57.59±6.85	0.948
LA diameter (mm)	46.91±5.14	47.20±5.31	46.45±4.94	0.599
LA indexed volume (ml/m^2^)	52.38±13.73	53.57±14.57	50.38±12.28	0.405
LAA flow speed (cm/s)	30.26±9.69	28.71±8.99	32.83±10.49	0.125
Spontaneous contrast	32 (56.1%)	20 (57.1%)	12 (54.5%)	1
LA thrombus	5 (8.8%)	4 (11.4%)	1 (4.5%)	0.639
Pre-ECV pro-BNP	1090 (595-1960)	1280 (565–2450)	870 (626-1344)	0.254
Pre-ECV CRP	0.61 (0.30–1.10)	0.50 (0.25–1.00)	1.10 (0.50–1.50)	0.070
Pre-ECV INR	2.73 (2.47–3.28)	2.73 (2.50–3.29)	2.63 (2.43–3.21)	0.948
Maximum F in V1 (mm)	1.11±0.51	1.41±0.41	0.64±0.16	<0.001
Post-ECV Morris Index	29 (58%)	21 (63.6%)	8 (47.1%)	0.366
Post-ECV P wave duration in lead II (ms)	128.41±26.42	130.38±20.54	124.35±36.16	0.542

Quantitative variables described as mean ± standard deviation or median (interquartile range); categorical variables described by frequency (percentage). *Association between cAF and fAF: t-Student test for independent samples or non-parametric Mann-Whitney test (quantitative variables); Fisher’s exact test (categorical variables); p < 0.05. cAF: coarse f-waves atrial fibrillation; fAF: fine f-waves atrial fibrillation; BMI: body mass index; CAD: coronary artery disease; HF: heart failure; PAD: peripheral artery disease; AF: atrial fibrillation; DOAC: direct oral anticoagulant; LVEF: left ventricular ejection fraction; LA: left atrium; LAA: left atrial appendage; ECV: electric cardioversion; BNP: brain natriuretic peptide; CRP: C reactive protein; INR: international normalized ratio.

### Laboratory and echocardiographic features

The mean LVEF was preserved. Only 7 patients (12.3%) had LVEF < 40%. Despite anticoagulation, thrombus and/or significant spontaneous contrast in the LA were observed in 35 patients. The mean values of pre-ECV pro-BNP and C-reactive protein (CRP) were high ( Table 1 ).

### Electrocardiographic characteristics

The amplitude of the f-max waves measured in V1 ranged from 0.3 to 2.9 mm. The Morris index was altered in most patients who had restored sinus rhythm, and the mean duration of the P wave in lead II in these patients was high ( Table 1 ).

### Features based on the amplitude of f waves

There were no differences between groups regarding clinical and echocardiographic characteristics, except for weight. The fAF group consisted of patients with higher weight values than the cAF group ( Table 1 ).

### ECV success

None of the parameters interfered with the success of ECV. Only the presence of cAF favored this outcome (94.3% vs. 72.7%, p = 0.036; OR 6.17; 95% CI 1.21-34.5) ( Table 2 and Figure 4 ).

**Table 2:** Association among echocardiographic, laboratory and electrocardiographic parameters with success in electric cardioversion

Variable	N (57)	Success N=49 (86%)	Failure N=8 (14%)	p*
Age (years)		62.55±10.40	55.25±12.22	0.088
Gender Female	17	15 (88.2%)	2 (11.8%)	
Male	40	34 (85%)	6 (15%)	0.748
Weight (Kg)		84.82±23.39	94.00±18.39	0.296
Body surface (m^2^)		1.94±0.29	2.07±0.23	0.244
BMI (Kg/m^2^)		29.46±6.03	31.65±6.17	0.344
Hypertension	47	42 (89.4%)	5 (10.6%)	0.126
Diabetes	14	12 (85.7%)	2 (14.3%)	0.975
CAD	9	9 (100%)	0 (0%)	0.332**
HF	4	4 (100%)	0 (0%)	1**
Stroke	7	6 (85.7%)	1 (14.3%)	0.977
PAD	4	4 (100%)	0 (0%)	1**
CHA_2_DS_2_VASc		2 (1–3)	1 (0.5–3)	0.200
0	5	3 (60%)	2 (40%)	
1	17	14 (82.3%)	3 (17.6%)	
≥ 2	35	32 (91.4%)	3 (8.6%)	
AF duration (days)		210 (100–370)	135 (75–270)	0.190
Amiodarone	54	48 (88.9%)	6 (11.1%)	-
Propafenone	3	1 (33.3%)	2 (66.7%)	-
Β-blocker	42	37 (88.1%)	5 (11.9%)	0.443
LVEF (%)		55.67±11.68	54±11.39	0.702
LA diameter (mm)		47.24±5.11	44.88±5.19	0.229
LA indexed volume (ml/m^2^)		52.67±14.12	50.29±11.32	0.665
LAA flow speed (cm/s)		29.43±8.81	35.25±13.54	0.125
Spontaneous contrast	32	29 (90.6%)	3 (9.4%)	0.262
LA thrombus	5	4 (80%)	1 (20%)	0.690
Pre-ECV pro-BNP		1195 (564–2005)	776 (649–1272)	0.607
Pre-ECV CRP		0.60 (0.25–1.10)	0.71 (0.55–1.50)	0.142
Pre-ECV INR		2.72 (2.50–3.29)	2.72 (2.24–2.95)	0.836
Maximum F in V1 (mm)		1.15±0.50	0.85±0.47	0.118
≥ 1 (cAF)	35	33 (94.3%)	2 (5.7%)	
<1 (fAF)	22	16 (72.7%)	6 (27.3%)	0.036

Quantitative variables described as mean ± standard deviation or median (interquartile range); categorical variables described by frequency (percentage). *Univariable logistic regression model and Wald test, p < 0.05. ** Fisher’s exact test, p < 0.05. BMI: body mass index; CAD: coronary artery disease; HF: heart failure; PAD: peripheral artery disease; AF: atrial fibrillation; LVEF: left ventricular ejection fraction; LA: left atrium; LAA: left atrial appendage; ECV: electric cardioversion; BNP: brain natriuretic peptide; CRP: C reactive protein; INR: international normalized ratio; cAF: coarse f-waves atrial fibrillation; fAF: fine f-waves atrial fibrillation.

**Figure 4:** Forest plot with OR and 95% CI of clinical, echocardiographic and electrocardiographic parameters related to success in electric cardioversion (univariable analysis). cAF: coarse f-waves atrial fibrillation; CRP: C reactive protein; LA: left atrium; LAA: left atrial appendage; LVEF: left ventricular ejection fraction; BMI: body mass index.

An operational curve was fitted to determine the best cutoff point for maximum f in V1 associated with the success of ECV. The value of 1.0 mm was the one with the best accuracy ( Figure 5 ).

**Figure 5:** ROC curve of maximum f amplitude in V1 as a successful predictor of electric cardioversion.

Patients with fAF received a median of 3 (IQR 2–3.5) shocks compared with 2 (IQR 1–3) in the cAF group (p = 0.019). When analyzing only those with successful ECV, the fAF group required a greater number of shocks to revert to sinus rhythm [3 (IQR 1–3) vs. 2 (IQR 1–2), p = 0.064] ( Figure 6 ). Likewise, the maximum and cumulative energies (sum of loads) used for reversion to sinus rhythm were higher in the fAF group [150J (IQR 150–200J) vs. 150J (IQR 100–150J), p = 0.027 and 320J (IQR 200–450J) vs. 200J (IQR 100–300J), p = 0.020; respectively] ( Figure 7 ).

**Figure 6:** Number of shocks delivered in each group (A) and number of shocks needed (B) for reversion to sinus rhythm in both groups. cAF: coarse f-waves atrial fibrillation; fAF: fine f-waves atrial fibrillation.

**Figure 7:** Maximum (A) and cumulative (B) energies necessary for reversion to sinus rhythm in both groups. cAF: coarse f-waves atrial fibrillation; fAF: fine f-waves atrial fibrillation.

In the multivariable analysis, the presence of cAF was associated with the success of ECV (B = 2.470, p = 0.026), regardless of age and BMI, favoring reversion to sinus rhythm by 11.8 times.

### Intra and interobserver variability

The calculation of intraobserver variability showed CCC and C.b of 0.90 and 0.98 for maximum f in V1, respectively. Likewise, the CCC and C.b values for interobserver variability were 0.90 and 0.98, respectively.

## Discussion

In the present study, the amplitude of f waves was not associated with the clinical, laboratory, and echocardiographic parameters suggestive of increased risk of thromboembolism. However, it contributed to the prediction of reversion to sinus rhythm using ECV.

Several factors that increase the risk of thromboembolism in patients with AF are related to each other, making individual analysis difficult as independent factors. In the studied sample, all patients were on full anticoagulation (most were on warfarin and had strict pre-ECV INR control). In the publications that proposed to evaluate the correlation between f amplitude and thromboembolism, no sample consisted of 100% of patients adequately anticoagulated. In the study conducted by Icen et al.,^7^ for example, 89% of the patients were using anticoagulants and reports of thromboembolic events were described in patients outside the anticoagulation range. In the study of Nakagawa et al.,^8^ only 54% of patients were on full anticoagulation. In the research carried out by Yamamoto et al.,^9^ only those with spontaneous contrast or thrombus were indicated for anticoagulation (75%). All these studies showed percentage differences in anticoagulant therapy between groups defined based on f amplitude.

Despite adequate anticoagulation in all patients in the sample, 56.1% had significant spontaneous contrast, and 8.8% had LA thrombus, showing that other mechanisms not treated by anticoagulation would still be present, increasing the risk of thromboembolism. Even so, there was no significant correlation between these findings and the amplitude of f waves, a fact also found by Nakagawa et al.^8^ Similarly, the presence of spontaneous contrast was not associated with f amplitude in the analysis by Yamamoto et al.;^9^ however, the authors reported a higher percentage of patients with LA thrombus and thromboembolic events in the fAF group, which can be explained by the lower percentage of patients on anticoagulation in this group during follow-up.

Contrary to these findings, Li et al.^10^ found a relationship between cAF and the presence of spontaneous contrast, LA thrombus and LAA dysfunction. Although both groups were more uniform concerning anticoagulant therapy, the authors did not report on differences in the CHA_2_DS_2_VASc score between them, which could influence the variation in thrombogenesis. In addition, there was a difference of one month between the performance of the TEE and the ECG, which may have contributed to the findings. In the present study, patients with cAF and fAF had similar values for CHA_2_DS_2_VASc, age, BMI and other clinical parameters. All were on anticoagulation, and the TEE and ECG were performed simultaneously.

In the present sample, patients with mitral stenosis were not included. The reason is that blood stasis caused by flow obstruction in the mitral valve predisposes to echocardiographic changes, which in this case would be more related to the obstructive factor itself than to the amplitude of the f waves. Particularly, patients with stenosis have dilated and hypertrophic LA, with increased atrial intracavitary pressure. Since patients with mitral stenosis are mostly of rheumatic etiology and present younger age and fewer comorbidities, despite being larger, the atria are less electrically remodeled, generating larger reentrant circuits, which are expressed by a more prominent resultant vector in the ECG (cFA).^11 - 13^

As for LA size, no significant differences were observed between the groups, and these findings agree with several other publications.^8 , 10 , 14 , 15^ This is because atrial dilation does not reliably reflect the degree of electrical, structural, and histological remodeling suffered by the atrium. In both groups, the values found for atrial diameter and volume were high, which reduced the influence of this variable on the f waves.

As for the evaluation of the LAA, we observed a reduction in the flow velocity of the LAA in both groups, however, without differences between them. The LAA contributes to atrial electrical and mechanical activity despite being a structure attached to the LA. Correlating its changes with the amplitude of f is a challenge since many factors can influence its performance, such as morphology, function (measured by flow velocity or ejection fraction), degree of fibrosis, and the area of the entry orifice.^16^

Li et al.^10^ showed a correlation between cAF in patients with NVAF, and low flow velocity in the LAA, results contradictory to those of Yamamoto et al.^9^ and Nakagawa et al.^8 ,^who showed an association with the fAF group. On the other hand, Blackshear et al.,^14^ when evaluating 53 patients involved in SPAF III, did not find a relation between the amplitude of f-waves and the flow velocity in the LAA, justifying the finding due to temporal discordance between the ECG and the TEE. In the present study, there was a satisfactory temporal correlation between these exams; even so, no significant association was demonstrated with the amplitude of f.

Likewise, clinical variables showed no association with f-wave amplitude. The groups contained patients with similar age, sex and CHA_2_DS_2_VASc score, providing greater homogeneity and reducing interference with other variables. Given that the amplitude of f translates information about atrial remodeling, we would expect patients with fAF to have higher CHA_2_DS_2_VASc scores, duration of AF, and older ages. In this context, the sample size may have been a limiting factor.

Among the comorbidities presented, hypertension was more prevalent in the fAF group (90.9% vs. 77.1%), in agreement with the findings of Yilmaz et al.^17^ and Icen et al.^7^ in patients with NVAF. BMI also tended to be higher in the fAF group, which may have been a confounding factor since this relationship has not been described in the literature.

Regarding laboratory data, in the fAF group, CRP values were higher, although not statistically significant. Given that this represents the presence of an inflammatory process and is related to the risk of stroke and prognosis in patients with AF, it is plausible to expect higher values in patients with fAF since they have more frequently remodeled atria as a result of multiple factors, including those that generate inflammation.^18^ On the other hand, pro-BNP levels were found to be high in both groups. This finding is frequent in patients with AF and acts as a marker of atrial heart disease, in addition to being indicative of a higher risk of stroke and death in this population.^19^

Similar to previous studies, the ECV success rate was 86%.^20 - 22^ In the study conducted by Zhao et al.,^20^ despite cAF being associated with higher rates of maintenance of sinus rhythm after 6 weeks of ECV (72% vs. 42%), there was no difference in the immediate success of the procedure between the groups (100% cAF vs. 94% fAF). However, data on mitral valve disease were not reported, which would justify the early recurrence of AF after ECV.^23 , 24^ In the present study, the presence of cAF was an independent predictor for immediate reversion to sinus rhythm. In addition to higher ECV success rates, the presence of cAF resulted in the need for fewer shocks and lower maximum and cumulative energy compared to fAF. This is relevant in clinical practice as it contributes as one more factor to deciding whether or not to indicate ECV in patients with persistent AF.

It is possible that cAF is related to the presence of more viable muscle in the atria that accommodate more organized reentry circuits, facilitating the cancellation of wave fronts through cardioversion. Age, type of arrhythmia and duration of AF, factors that influence the success rates of ECV,^25^ did not influence the discriminatory power of the amplitude of f waves because they did not differ between the groups formed.

As for the lead analyzed, we chose V1 because it is the one that most expresses changes in the atria due to proximity, for presenting higher values of the f amplitude, facilitating measurement, and for having been the lead applied by most studies published on the topic since 1966.

As for the cutoff point used to classify AF, we chose the value of 1.0 mm based on the fact that there is no significant difference between the findings when using the value of 0.5 mm and 1 mm, as demonstrated by Peter et al. and the highest value facilitates its measurement.^5^ Using smaller cutoff points implies more accurate measurement techniques and more measurement errors, with few gains in sensitivity and specificity.

The use of antiarrhythmic drugs as a pre-treatment before ECV was allowed for better stabilization of atrial electrical activity and to prevent early recurrence of arrhythmia.^24^ The fact that almost all of them used amiodarone reduces the interference between the groups in the ECV result. In addition, Nault et al.^26^ demonstrated that antiarrhythmic drugs such as amiodarone did not influence the f amplitude.

It is possible that the small sample size may have influenced the results, particularly regarding the association between echocardiographic parameters and f amplitude. Studies with a greater number of patients are needed to establish these relationships.

## Conclusions

The amplitude of f was not associated with clinical and echocardiographic changes that signal a higher risk of thromboembolism. Maximum f wave ≥ 1.0 mm measured in lead V1 was associated with a higher chance of success in restoring sinus rhythm through ECV in patients with persistent NVAF. A greater number of shocks and energy was required for reversion to sinus rhythm in patients with fAF compared with cAF.
